# Systematic Review of Clinical and Pathophysiological Features of Genetic Creutzfeldt–Jakob Disease Caused by a Val-to-Ile Mutation at Codon 180 in the Prion Protein Gene

**DOI:** 10.3390/ijms232315172

**Published:** 2022-12-02

**Authors:** Taiki Matsubayashi, Nobuo Sanjo

**Affiliations:** Department of Neurology and Neurological Science, Tokyo Medical and Dental University Graduate School of Medical and Dental Sciences, 1-5-45 Yushima Bunkyo-ku, Tokyo 113-8510, Japan

**Keywords:** genetic prion disease, prion protein gene, genetic Creutzfeldt–Jakob disease, Val-to-Ile substitution at codon 180, normal prion proteins, pathological prion proteins, V180I

## Abstract

Genetic Creutzfeldt–Jakob disease (gCJD) is a subtype of genetic prion diseases (gPrDs) caused by the accumulation of mutated pathological prion proteins (PrP^Sc^). gCJD has a phenotypic similarity with sporadic CJD (sCJD). In Japan, gCJD with a Val to Ile substitution at codon 180 (V180I-gCJD) is the most frequent gPrD, while the mutation is extremely rare in countries other than Japan and Korea. In this article, we aim to review previously elucidated clinical and biochemical features of V180I-gCJD, expecting to advance the understanding of this unique subtype in gCJD. Compared to classical sCJD, specific clinical features of V180I-gCJD include older age at onset, a relatively slow progression of dementia, and a lower positivity for developing myoclonus, cerebellar, pyramidal signs, and visual disturbance. Diffuse edematous ribboning hyperintensity of the cerebral cortex, without occipital lobes in diffusion-weighted magnetic resonance imaging, is also specific. Laboratory data reveal the low positivity of PrP^Sc^ in the cerebrospinal fluid and periodic sharp wave complexes on an electroencephalogram. Most patients with V180I-gCJD have been reported to have no family history, probably due to the older age at onset, and clinical and biochemical features indicate the specific phenotype associated with the prion protein gene mutation.

## 1. Introduction

Prion diseases (PrDs) are fatal and transmissible neurodegenerative diseases caused by the extracellular deposition of aggregated misfolded pathological prion proteins (PrP^Sc^), which differ from normal prion proteins (PrP^C^) in that they have conformational changes and are abnormally misfolded [1]. The deposition and accumulation of PrP^Sc^ in the central nervous system lead to rapidly progressive neuronal dysfunction and neurodegeneration [2]. Human PrDs are classified into three major phenotypes based on PrP^Sc^ etiology: sporadic, genetic, and acquired (infectious) [3,4]. Genetic PrDs (gPrDs) result from mutations in the prion protein gene (*PRNP*) on chromosome 20. In 1930, Meggendorfer reported the first familial aggregation of PrD in German kindred (known as the “Backer” family) [5]. To date, over sixty pathological *PRNP* mutations have been identified [6]. A point mutation is the most frequent genetic type in the *PRNP* mutations, while octapeptide repeat insertions or deletions have rarely been reported [7]. gPrDs are divided into three subtypes based on their clinical, genetic, and pathological characteristics: genetic Creutzfeldt–Jakob disease (gCJD), Gerstmann–Sträussler–Scheinker disease, and fatal familial insomnia [8]. gPrD accounts for approximately 10–15% of human PrD cases worldwide (10.2% in western countries and 16.7% in Japan) [9,10,11], and the distribution and ratios of *PRNP* mutations differ greatly among countries [6,12]. For instance, the most frequent mutation in Japan is Val-to-Ile substitution at codon 180 (V180I; 41.2%), followed by Pro to Leu substitution at codon 102 (P102L), and Glu to Lys substitution at codon 200 (E200K), while E200K is the most common mutation in Europe [9,13].

The first patient with a V180I gCJD phenotype (V180I-gCJD) was reported in 1993 [14]. This mutation is now the most frequent in Japan and Korea, whereas it is extremely rare in other countries [9,15,16,17,18,19]. In addition to the skewed epidemiological localization and distribution, clinical features of patients with V180I-gCJD have been described as specific and different from those with sporadic CJD (sCJD) and other gPrDs in some aspects [20]. The Japanese Prion Disease Surveillance Committee conducted a large cohort clinical study involving 186 Japanese patients with V180I-gCJD and clarified the detailed clinical features of V180I-gCJD compared to sCJD [21]. Most patients with V180I-gCJD had no familial history, and the penetrance of the mutation was estimated as very low, approximately 1% [22]. Furthermore, a Korean population study identified V180I mutation in a few non-CJD individuals [23]. Thus, a few researchers questioned the pathogenicity of the variant [23]. However, we recently reported the detailed biochemical features of V180I-gCJD using autopsied brain samples, indicating the pathogenicity of the V180I *PRNP* mutation [24]. In this article, we aimed to review the previously elucidated clinical and biochemical features of V180I-gCJD, expecting to advance the understanding of this unique subtype in gCJD. The following clinical features were specific to V180I-gCJD: an extremely low frequency of family history; a late onset age and slow progression; a low frequency of myoclonus, visual disturbance, and cerebellar signs; a low positive rate of periodic sharp wave complexes (PSWCs) on electroencephalogram (EEG); a predominant involvement of the cerebral cortex in diffusion-weighted imaging (DWI) sequences of magnetic resonance imaging (DWI-MRI) and single photon emission computed tomography (SPECT) imaging, and an absence of the occipital lobes, posterior to the parieto-occipital sulcus, brainstem, or the cerebellum; a relatively low positive rate of PrP^Sc^ via cerebrospinal fluid (CSF) real-time quaking-induced conversion (RT-QuIC); and a high positive rate of 14-3-3 protein and t-tau protein in CSF examination.

## 2. Material and Methods

**Figure 1:**
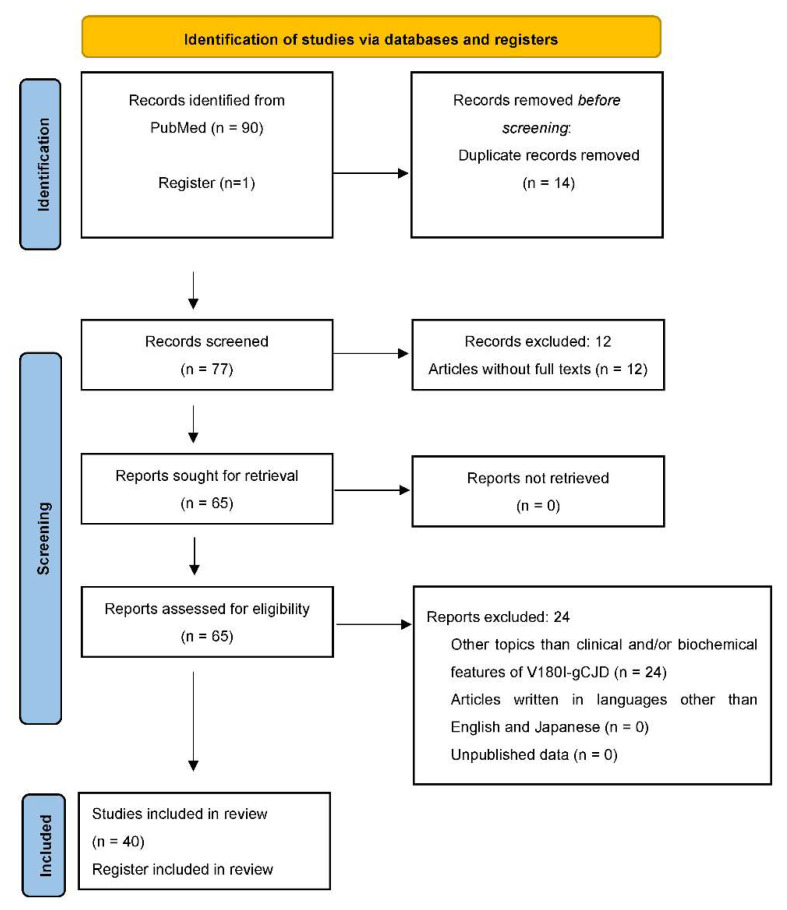
PRISMA flow-chart diagram showing the paper selection process.

This systematic review was conducted according to the Preferred Reporting Items for Systematic Reviews and Meta-Analyses (PRISMA) statement in the flowchart below (Figure 1). For the PubMed search dated August 2022, three search strategies were developed: 1. V180I [Title/Abstract], 2. Valine [Title/Abstract] AND Isoleucine [Title/Abstract] AND codon 180 [Title/Abstract], 3. CJD [Title/Abstract] AND codon 180 [Title/Abstract] AND point mutation [Title/Abstract]. Additionally, a record was identified from a register (Japanese Prion Disease Surveillance Committee). For inclusion criteria, only articles describing clinical and/or biochemical features of V180I-gCJD were considered. The exclusion criteria were as follows: articles written in languages other than English and Japanese, unpublished data, articles without full texts, and studies unrelated to clinical and/or biochemical features of V180I-gCJD including genetic features. Articles written in Japanese were analyzed because almost all patients with V180I-gCJD have been reported from Japan. All authors (T.M, N.S) with expertise in medical evaluations and in research methodology independently screened titles, abstracts, and full texts for eligibility, assessed generalizability, and collected data from eligible articles. The data of finally included and referred to articles in this review are synthesized and summarized in Table 1.

## 3. Results and Discussion

### 3.1. Clinical Features of V180I-gCJD

#### 3.1.1. Clinical Features

##### Epidemiological Factors of V180I-gCJD

In our large cohort study, including 186 Japanese patients with V180I-gCJD, the number of female patients was greater than that of males (108 vs. 78) [21], indicating a possible sexual effect on the incidence of V180I-gCJD and other mutations in gPrDs; women appear more susceptible [9]. Concerning family history, 11 of the 186 patients (5.9%) had a family member diagnosed with dementia. In total, 3 of the 11 patients had a family history of CJD [21]. The low frequency of family history in V180I-gCJD is consistent with previous studies [10,20,25]. Methionine homozygosity at *PRNP* codon 129 was observed in most patients with V180I-gCJD (V180I-MM gCJD; 75.5%), while methionine/valine (MV) heterozygosity was observed in others (V180I-MV gCJD; 24.5%). In contrast, all patients with V180I-gCJD who tested for the codon 219 polymorphism had glutamic acid homozygosity [21]. Regarding codon 129 polymorphism, the frequency of MV heterozygosity in patients with V180I-gCJD was higher than in the general Japanese population. This is possibly explained by a case-control study in Japan indicating that methionine homozygosity was protective against gCJD, including V180I mutation, but predisposing to sCJD [51]. Concerning codon 219 polymorphism, previous studies have reported the possibility of its protective activity against sCJD [52,53], and the high frequency of glutamic acid homozygous in V180I-gCJD suggests that heterozygosity would also be a protective factor in V180I-gCJD.

According to the recent Japanese Prion Disease Surveillance Committee data (September 2022), the average age at the onset of V180I-gCJD is 78.9 ± 7.0 years, which is approximately 10 years older than that of sCJD with methionine homozygosity and type 1 PrP^Sc^ (MM1) [21]. Moreover, in a retrospective study including the major genotypes of gPrDs in Japan, we reported that the onset age of V180I-gCJD was the highest among all types of gPrDs. The positive rate of CSF PrP^Sc^ in V180I using RT-QuIC is the lowest among these mutations, possibly due to the slow progression during the preclinical stage and the older age at onset [26]. The oldest age at onset is probably associated with low positivity of family history due to the longevity of the mutation carriers [16,54]. In our cohort study, the average period between disease onset and death in V180I-MM gCJD was longer (23.1 ± 15.1 months) than in sCJD-MM1 (17.2 ± 12.5 months); this indicates a relatively slow progression [21]. Some patients with V180I-gCJD have been reported to survive for a long period, up to 198 months (16.5 years). This may be due to tube feeding or very mild brainstem involvement, proven by pathological analysis and imaging biomarkers [19,27,28,29,30]. 

##### Clinical Symptoms and Signs of V180I-gCJD

The most common clinical symptom of V180I-MM gCJD was cognitive impairment (100%), followed by akinetic mutism (54%), extrapyramidal signs (53.4%), psychiatric symptoms (52.3%), and pyramidal signs (50%) [21]. In contrast, myoclonus, one of the classic triads of classic sCJD, was significantly less frequent in V180I-MM gCJD (35.4%) than in sCJD-MM1 (88.1%) [21]. Furthermore, visual disturbance and cerebellar signs were identified in 8.3% and 34%, respectively, in our cohort study [21]. Additionally, the period between disease onset and the occurrence of myoclonus, cerebellar signs, visual disturbance, and akinetic mutism in V180I-MM gCJD, was significantly longer than that in sCJD-MM1 [21]. As stated later in this article, low frequency and the slow development of visual disturbance and cerebellar signs in V180I-gCJD may be associated with the preserved occipital lobes and cerebellum in neuropathological examination and brain imaging. Regarding the codon 129 polymorphism, clinical features did not differ significantly between V180I-MM and V180I-MV gCJD [21]. 

Cerebral cortical dysfunctions, such as disorientation, dementia, and aphasia, have been reported as major initial symptoms of V180I-gCJD [20,21,27,31,32,33]. Additionally, neuropsychiatric symptoms, such as abnormal behavior, pathological laughing, persecution delusion, wandering, motivation loss, and person delusional misidentification, have been reported as initial manifestations [33,34,35,36].

Dopaminergic denervation in the basal ganglia causes extrapyramidal signs, such as rigidity of the extremities and akinesia [55], and extrapyramidal signs in V180I-gCJD have been described in several cases [18,19,31,33,37,38,39,40]. Pathological studies revealed spongiform changes in the basal ganglia and PrP deposition in some patients with V180I-gCJD [27,31]. Levodopa, mainly used to treat Parkinson’s disease [56], was ineffective on extrapyramidal symptoms in V180I-gCJD [38,39], indicating postsynaptic dysfunction of the nigrostriatal pathway. Interestingly, previous case reports described two patients with V180I-gCJD who were misdiagnosed as having Lewy body disease due to extrapyramidal signs and the positivity of dopamine transporter (DAT)-single photon emission computed tomography (SPECT). A patient with cognitive impairment and DAT-SPECT positivity, which reflects the degree of presynaptic dopaminergic neuron loss, indicating a bilateral reduction in tracer binding in the basal ganglia, was initially diagnosed with dementia with Lewy bodies [39]. 

Despite the lack of a definition, patients with akinetic mutism generally do not show any voluntary movement (akinesia) or speech (mutism) [57]. In previous case reports, several patients with V180I-gCJD who had reached akinetic mutism could orally consume foods, probably using their autonomic reflex swallowing [30,33,41,42]. One patient with akinetic mutism had a well-preserved swallowing function, especially the brainstem-swallowing reflex [30].

#### 3.1.2. EEG

The most typical EEG finding in CJD is PSWCs, symmetrical generalized triphasic, biphasic, or mixed complexes occurring approximately every second [10]. For sCJD, PSWCs had a sensitivity of 67% and a specificity of 86% [58]. However, the sensitivity of PSWCs on EEG in gPrDs is lower than in sCJD [10], and the sensitivity in gPrD differs among *PRNP* mutations [26]. PSWC positivity in V180I-gCJD was quite low (9.3%), and its rate was significantly lower than that in patients with sCJD-MM1 [21]. Most patients with V180I-gCJD had background slowing on EEG [27]. However, the low frequency of PSWCs and the lack of myoclonus in the early stage may make distinguishing V180I-gCJD from other neurodegenerative diseases with progressive dementia difficult [25].

#### 3.1.3. Brain Imaging

Magnetic resonance imaging (MRI) is a reliable, non-invasive diagnostic tool for PrDs. DWI-MRI detects protons in water molecules during gradient pulse application in three orthogonal directions [59]. Additionally, DWI-MRI is superior to fluid-attenuated inversion recovery in CJD diagnosis [60]. The hyperintensity patterns in DWI-MRI depend on the subtypes of PrDs [61]. For example, DWI-MRI of sCJD-MM1 shows signal hyperintensity in the bilateral asymmetric cerebral cortex (cortical-ribboning) and/or basal ganglia [62]. In a large cohort study, the characteristic signal hyperintensity change on DWI-MRI had a sensitivity of 92% and specificity of 97% for sCJD [62]. 

Typical MRI features of V180I-gCJD include [20,25,43,44]:

1. bilateral cortical signal hyperintensity on DWI-MRI with or without basal ganglia involvement,
2. cortical ribboning edematous swelling on fluid-attenuated inversion recovery and T2-weighted images,
3. absence in the occipital lobes, posterior to the parieto-occipital sulcus, brainstem, or cerebellum until the terminal stage.

The sensitivity of signal hyperintensity on DWI-MRI in V180I-MM gCJD was high (99.3%) and similar to that in sCJD-MM1 [21], suggesting that DWI-MRI is effective for the early diagnosis of V180I-gCJD. Notably, DWI-MRI revealed cortical signal hyperintensity in three patients with V180I-gCJD before the onset of clinical symptoms [40,44,45]. Pathophysiologically, it has been debated whether signal hyperintensity on DWI-MRI correlates with brain function in V180I-gCJD, because the absence of signal hyperintensity in the cerebellum and occipital cortex on DWI-MRI and the low frequency of cerebellar and visual symptoms are typical clinical features of V180I-gCJD. In V180I-gCJD, signal hyperintensity on DWI-MRI is assumed to reflect the spongiform change, especially various-sized and nonconfluent vacuoles, rather than PrP deposition [25,32]. An autopsy case of V180I-gCJD, without cerebellar or visual symptoms, had neither signal hyperintensity in the cerebellum and occipital lobes on DWI-MRI, nor spongiform changes in the cerebellum and medial occipital cortex in neuropathological examinations [46]. Hence, DWI-MRI findings in V180I-gCJD may correlate with brain function. Patients with V180I-gCJD have stable and larger brain vacuole sizes, regardless of disease duration, than patients with sCJD-MM1 [27]. The correlation between the signal strength of hyperintensity on MRI and pathological features of the brain could be clarified in future clinicopathological studies. 

SPECT imaging can map and measure the brain’s relative regional cerebral blood flow using an intravenously injected radiolabeled tracer [63]. In V180I-gCJD, SPECT imaging revealed marked cerebral blood flow reduction, predominantly in cerebral cortical regions corresponding to brain areas with signal hyperintensity on DWI-MRI [25,33,37,43].

A serial imaging study, including three cases with V180I-gCJD, discovered that signal hyperintensity on DWI-MRI and decreased regional cerebral blood flow on SPECT became widespread as the disease progressed [37]. Thus, these brain imaging biomarkers in V180I-gCJD may have clinical importance for monitoring disease progression and treatment response in therapeutic trials and diagnosis.

#### 3.1.4. CSF Biomarkers

##### RT-QuIC

The development and clinical application of RT-QuIC, a PrP^Sc^ amplification assay, has been a breakthrough for the accurate premortem diagnosis of PrDs [64]. In this assay, the aggregation reaction (amyloid fibril formation) of recombinant PrP, which has the same structure as PrP^C^, is continuously induced by patient-derived PrP^Sc^ and used as a seed for the reaction in vitro, resulting in PrP^Sc^ amplification [65]. Prospective studies, including sCJD as cases and rapidly progressive dementia as controls, have reported that the specificity of RT-QuIC in the CSF for sCJD was 99–100% [66,67,68,69]. Hence, RT-QuIC was adopted in the newly proposed diagnostic criteria for sCJD in 2021 [64]. 

We previously reported that the positive rate of PrP^Sc^ via CSF RT-QuIC in patients with V180I-MM gCJD (67.9%) was significantly lower than in patients with sCJD-MM1 (90%) [21], probably due to the diverse molecular sizes and/or conformations of PrP^Sc^ produced by the V180I mutation [24]. Autopsy analysis of patients with V180I-gCJD revealed very weak immunoreactivity to abnormal PrP in western blotting and immunohistochemistry [26,27,31,32,46]. This indicates that low levels of PrP accumulation in some areas of the brain, or lower specificity of anti-PrP (3F4) to the V180I mutant PrP^Sc^, may result in the low positive rate for the RT-QuIC assay. Regarding the *PRNP* codon 129 polymorphism, although almost all V180I mutations exist on the same methionine allele, patients with V180I-MV gCJD showed a significantly lower positive RT-QuIC ratio of PrP^Sc^ in the CSF than patients with V180I-MM gCJD [21]. The codon 129 polymorphism may contribute to the difference in PrP^Sc^ positivity between V180I-MM and V180I-MV because it is important in determining the disease phenotype in gPrDs [70,71,72,73]. 

##### 14-3-3 Protein

14-3-3 protein is abundantly expressed in the brain and localized in the cytoplasm and cell membrane, where it is involved in various functions such as cell signaling, cell growth, and apoptosis [74]. 14-3-3 protein was first reported as a highly specific CSF biomarker for CJD [75] and 14-3-3 protein detection via western blot was referred to in the World Health Organization criteria for sCJD [76]. However, later studies revealed a wide range of specificity in the diagnosis of CJD (40–92%) [77,78], and increased 14-3-3 protein was observed in other neurodegenerative dementia (such as Alzheimer’s disease, vascular dementia, Lewy body disease, frontotemporal dementia, Parkinson’s disease, and encephalitis) [78]. 

In our cohort study, the positive rate of 14-3-3 protein in the CSF did not differ significantly between V180I-gCJD (80.2%) and sCJD older than 75 years (87.5%). Similarly, the positive rate in V180I-MM gCJD (86.8%) was approximately as high as that in sCJD-MM1 (87.1%) [21]. In our retrospective study comparing various subtypes of gPrDs, high CSF 14-3-3 and tau protein levels were detected in patients with V180I, E200K, and M232R (rapid-type) gCJD. Increased 14-3-3 protein levels in V180I-gCJD indicated the severity of the pathological process and its associated neuronal damage. Regarding the codon 129 polymorphism, a significantly lower positive rate of 14-3-3 proteins was discovered in V180I-MV gCJD than in V180I-MM gCJD [21].

##### Tau Protein

Tau is a microtubule-associated protein expressed in neurons and glial cells that aids microtubule binding and stabilization [79]. Increased CSF total tau (t-tau) proteins were discovered in patients with sCJD [80]; however, lower specificity and increased CSF t-tau were also identified in neurodegenerative diseases other than sCJD [77].

We previously reported that the ratio of t-tau proteins in the CSF of V180I-gCJD [21] was 85.9%, which did not differ significantly from that in sCJD. Positive rates of t-tau in V180I-MM gCJD (90.6%) and sCJD-MM1 (87.1%) were similar, and increased levels of t-tau proteins and 14-3-3 proteins are likely to reflect the severity of brain damage [26]. V180I-MV gCJD had a significantly lower level of t-tau proteins in the CSF than V180I-MM gCJD. Moreover, the difference in CSF t-tau protein in the codon 129 polymorphism was observed in PrP^Sc^ and 14-3-3 protein positivity [21].

### 3.2. Biochemical Features of V180I-gCJD

#### 3.2.1. Neuropathological Examination

##### Neuropathological Findings of sCJD and gCJD

A neuropathological examination is the only tool for making a definite diagnosis of PrDs; furthermore, neuropathological findings are critical in determining clinical phenotypes of PrDs [81,82]. In sCJD, the clinicopathological phenotypic variability is largely influenced by the *PRNP* polymorphism at codon 129 and the prion strain type (PrP^Sc^ types) [83]. The *PRNP* codon 129 encodes either methionine (M) or valine (V), and western blot analysis reveals that the unglycosylated protease K-resistant C-terminal core of type 1 and type 2 PrP^Sc^ is 21 and 19 kDa, respectively. Based on the combination of the codon 129 polymorphism (MM, MV, or VV) and the type of PrP^Sc^ (type 1 or type2), sCJD has been divided into six subtypes: MM1, MM2, MV1, MV2, VV1, and VV2 [84]. Neuropathological findings of sCJD, particularly the most common MM1 subtype in Japan, are characterized by spongiform changes in the gray matter, astrogliosis, neuropil rarefaction, neuronal loss, and PrP deposition. The spongiform changes consist of fine vacuoles and homogeneous distribution in sCJD-MM1, and PrP immunostaining reveals diffuse granular synaptic type PrP deposition [85,86]. The detailed features of neuropathological findings vary according to the *PRNP* mutations [7].

##### Neuropathological Findings of V180I-gCJD

In contrast with diffuse cerebral atrophy in macroscopic analysis, relatively preserved parenchyma was identified in the cerebellum, brainstem, precentral gyrus, and hippocampus in V180I-gCJD. Depigmentation was not apparent in the substantia nigra or the locus coeruleus [27,28,31,32,46,47]. In microscopic analysis, the most characteristic feature was an extensive spongiform change in the cerebral cortex [24,85]. Interestingly, the V180I-gCJD occipital cortex of patients with V180I, especially some areas in the medial occipital lobe, was well preserved from spongiform changes compared to the other cortices [28,46,47]. Gliosis and neuronal loss were also observed in the cerebral cortices; however, the degree was relatively mild compared to sCJD [27,31,32]. Spongiform changes ranged from mild to severe in the striatum and were relatively mild in the hippocampus and thalamus [24,27,28,31,32,46,47]. The cerebellum was generally preserved [24,28,32,34,35,46,47], except for a few cases that showed mild spongiform changes, particularly in molecular layers [27,31]. In the brainstem, spongiform changes, neuron loss, and gliosis were not observed [24,28,32,35,46,47,48]. Furthermore, no apparent correlation was observed between total disease duration and the severity of spongiform changes in the cerebral cortex and hippocampus [24]. Patients with V180I-gCJD had severe spongiform changes in the cerebral cortex similar to those with sCJD-MM1, regardless of the disease duration [87]. Moreover, the spongiform changes consisted of various-sized and non-confluent vacuoles [33]. The vacuole sizes and their dispersion in V180I-gCJD were larger than those in sCJD-MM1 [27].

Neurofibrillary tangles, senile plaques, and amyloid-β deposition are the pathological hallmarks of Alzheimer’s disease. These biomarkers were identified in some patients with V180I-gCJD [24,31,32,46,47]. Concerning Aβ plaque, Aβ deposits detected in the cerebral cortex were composed of Aβ40 rather than Aβ42 [24]. As previously stated, the aging pathology in V180I-gCJD may be associated with relatively older age at onset. In contrast, Lewy bodies and α-synuclein pathology were absent [31,32,46].

Immunochemical staining for PrP revealed diffuse synaptic-type PrP deposition, mainly in the cerebral cortex; however, the immunoreactivity using 3F4 antibody was extremely weak [24,28,32,46,47]. The cerebellum is preserved from PrP deposition [24,32,35,46,47], although a patient with both PrP deposition and spongiform changes limited to the molecular layer of the cerebellum was reported [31]. No PrP deposition has been observed in the brainstem so far [24,28,32,35,46,47].

#### 3.2.2. Western Blot Analysis and Pathogenicity of V180I Mutation

**Figure 2:**
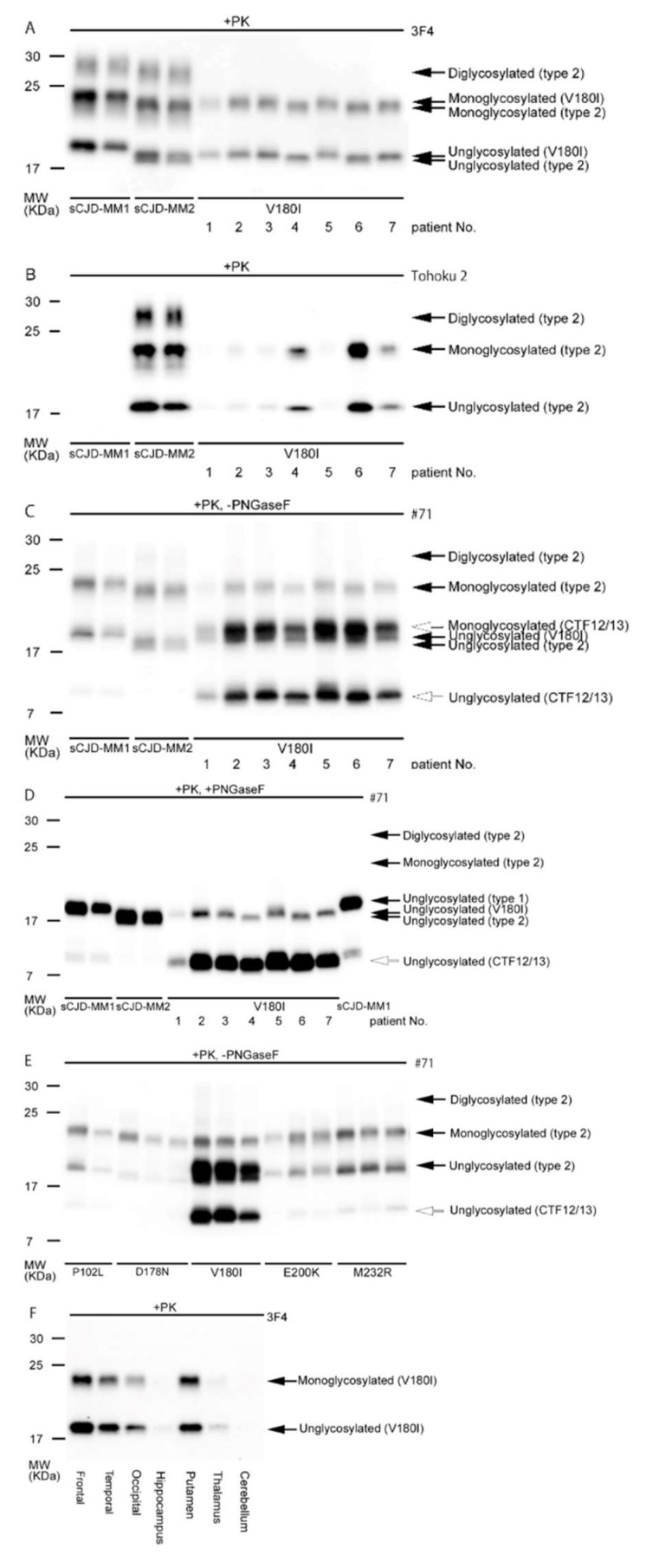
Western blot analysis of PrP^res^ in patients with V180I-gCJD. (**A**–**D**): Brain homogenates from seven patients with V180I-gCJD, two patients with sCJD-MM1, and two patients with sCJD-MM2C were treated with proteinase K and SDS-PAGE. The figure shows the results of analyses with 3F4 (**A**), the type-2 specific antibody Tohoku 2 (**B**), and the carboxyl terminal-specific antibody #71 (**C**). In subsequent analyses with the #71 antibody, the samples were treated with both proteinase K and PNGase F (**D**). (**E**) Quantitative analysis of the ratio of CTF12/13 to the major unglycosylated form of PrP^res^ with V180I, relative to that observed in other forms of gCJD (P102L, D178N, E200K, M232R). (**F**) Relative amount of PrP^res^ in various brain regions (Frontal: frontal neocortex, Temporal: temporal neocortex, Occipital: occipital neocortex, Cerebellum: cerebellar cortex). The same amount of brain homogenate was loaded on the SDS-PAGE after treatment with proteinase K. Among the three neocortical samples, the frontal cortex exhibited the greatest amount of PrP^res^, while the occipital cortex exhibited least. Very small amounts of PrP^res^ were observed in the hippocampus, thalamus, and cerebellum. This Figure was cited from reference [24]. (**A**–**D**): lanes 1–2, sCJD-MM1; lanes 3–4, sCJD-MM2C; lanes 5–11, V180I-gCJD; lane 12, sCJD-MM1. PrP^res^, protease-resistant abnormal prion proteins; V180I, valine to isoleucine substitution at codon 180; PNGase F, N-glycosidase F; SDS-PAGE, sodium dodecyl sulfate-polyacrylamide gel electrophoresis; gCJD, genetic Creutzfeldt–Jakob disease; sCJD, sporadic Creutzfeldt–Jakob disease; CTF, carboxyl terminal fragments.

**Figure 3:**
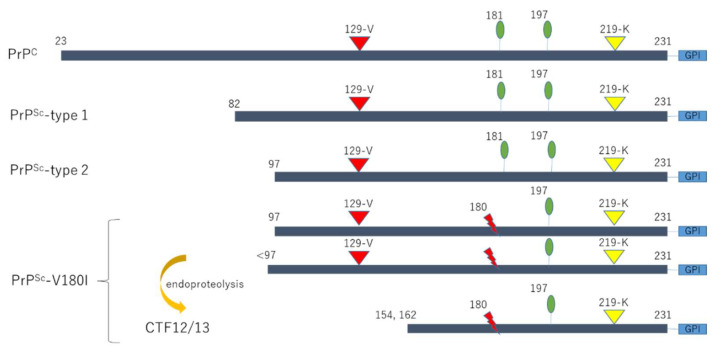
Diagram of the pathogenic PrP fragments. Two glycosylated sites are represented as green ellipses. Major PrP^Sc^ fragments (types 1 and 2) are shown, along with the two components of the PrP^Sc^-V180I fragments. The polymorphisms in the prion gene are represented as inverted triangles; additionally, red (129-V) and yellow (219-K) inverted triangles show exacerbating and protective effects, respectively, on PrP^Sc^-V180I toxicity [51]. This Figure was modified and cited from references [24,26]. PrP, prion proteins; PrP^C^, normal prion proteins; PrP^Sc^, abnormal prion proteins; PrP^res^, protease-resistant abnormal prion proteins; V180I, valine to isoleucine substitution at codon 180; 129-V, valine polymorphism at codon 129; 219-K, lysine polymorphism at codon 219.

The western blot analysis of PrDs reveals three distinct bands depending on the glycosylation of PrP^Sc^, which has two potential glycosylation sites at codons 181 and 197, corresponding to the di, mono, or unglycosylated modifications of the protein [83]. Our previous study analyzed the biochemical features of V180I-gCJD using brain samples from seven autopsied patients [24]. We revealed that unglycosylated forms of PrP^Sc^ in patients with V180I-gCJD were classified into two types based on molecular weight differences in western blot analysis, using antibodies against the amino-terminus. Namely, one band was detected at slightly higher levels than those of type 2 PrP^Sc^ in four out of seven patients (Figure 2A: patient 1–3, 5), while another was consistent with type 2 PrP^Sc^ in the remaining patients (Figure 2A,B: patient 4, 6, 7), indicating that amino-terminal length varies according to cases via unknown mechanisms. In contrast, the diglycosylated form of PrP^Sc^ was not observed, probably because the mutation at codon 180 prevented the diglycosylated and monoglycosylated forms at codon 181 from converting into PrP^Sc^ [49,50]. In contrast, carboxyl terminal-specific antibodies verified the presence of two carboxyl-terminal fragments (CTF12/13) [88]: one band at a molecular weight of approximately 11 kDa, which could be the unglycosylated CTF12/13 form, and another band at a molecular weight slightly greater than that of the unglycosylated form of PrP^Sc^, which could be the monoglycosylated CTF12/13 form (Figure 2C,D). Three components of the PrP^Sc^-V180I fragment complex and major PrP^Sc^ fragments (types 1 and 2) were also shown as a diagram (Figure 3). Furthermore, the amount of unglycosylated CTF12/13 in patients with V180I-gCJD was higher than that in patients with other gPrDs (Figure 2E).

Regarding brain regions, the neocortices accumulated more PrP^Sc^ than the hippocampus (Figure 2F), corresponding to the distribution of spongiform change, which was extensive in the neocortices and relatively mild in the hippocampus. These findings indicate that PrP^Sc^ accumulation in the neocortex is associated with neurotoxicity, leading to severe spongiosis. Therefore, these pathological findings indicate that the V180I mutation is probably pathogenic, not a polymorphism.

## 4. Conclusions

The following clinical features were specific to V180I-gCJD:

1. an extremely low frequency of family history;
2. a late onset age and slow progression;
3. major initial symptoms of cerebral cortical dysfunctions and a low frequency of myoclonus, visual disturbance, and cerebellar signs;
4. a low positive rate of PSWCs on EEGs;
5. a predominant involvement of the cerebral cortex in DWI-MRI and SPECT imaging, and the absence of abnormal findings in occipital lobes, posterior to the parieto-occipital sulcus, brainstem, or the cerebellum;
6. a relatively low positive rate of PrP^Sc^ by CSF RT-QuIC, and a high positive rate of 14-3-3 protein and t-tau protein in CSF examination.

Low frequency of family history and the low penetrance of the V180I may be associated with a late onset age and slow progression of the disease. Namely, the carriers of the mutation probably die before the development of the disease. Furthermore, we speculate that the low positive rate of PrP^Sc^ by CSF RT-QuIC results in the slow progression during the preclinical stage, leading to a late onset age. Cerebral cortical dysfunctions are major initial symptoms probably because the cerebral cortex is predominantly involved in both imaging biomarkers and neuropathological examinations. Similarly, the frequency of visual disturbance and cerebellar signs is low probably due to the absence of abnormal findings in the occipital lobes and the cerebellum.

Recently, remarkable progress has been made in the therapeutic development of PrDs. Thus, early diagnosis is indispensable for initiating effective treatment.

In biochemical features, extensive spongiform change in the cerebral cortex is the most prominent neuropathological finding. Immunohistochemistry for PrP deposition shows diffuse synaptic type, mainly in the cerebral cortex; however, the immunoreactivity is extremely weak. Western blot analysis revealed that the amount of accumulated PrP^Sc^ was greater in the cerebral neocortices, where severe spongiform change was observed, than in the hippocampus, where milder spongiform change was observed. These findings indicate that PrP^Sc^ accumulation in the neocortex is associated with severe neurotoxicity, leading to spongiosis.

The development of diagnostic biomarkers and therapeutic progress of PrDs has been promoted. However, future clinical practices of PrDs, including V180I-gCJD, will require establishing a cohort system and a multicenter network as a framework for conducting clinical trials to connect diagnosis and treatment.

## Figures and Tables

**Table 1:** Articles analyzed and referred to in this review.

Article *	Dataset Size **
Nozaki et al. 2010 [10]	89
Yang et al. 2010 [16]	1
Shi et al. 2014 [18]	1
Ryoo et al. 2022 [19]	1
Jin et al. 2004 [20]	9
Qina et al. 2014 [21]	186
Ito et al. 2018 [24]	7
Kutsukura et al. 2009 [25]	3
Higuma et al. 2013 [26]	151
Akagi et al. 2018 [27]	6
Hayashi et al. 2020 [28]	1
Nomura et al. 2020 [29]	1
Kunieda et al. 2020 [30]	1
Iwasaki et al. 2011 [31]	1
Iwasaki et al. 2017 [32]	1
Iwasaki et al. 2019 [33]	1
Suzuki et al. 2008 [34]	1
Iwasaki et al. 2012 [35]	3
Nagata et al. 2022 [36]	1
Hayashi et al. 2016 [37]	3
Fujita et al. 2021 [38]	1
Tomizawa et al. 2020 [39]	1
Koizumi et al. 2021 [40]	1
Deguchi et al. 2012 [41]	1
Iwasaki et al. 2017 [42]	3
Kono et al. 2011 [43]	1
Terasawa et al. 2012 [44]	1
Suzuki et al. 2022 [45]	1
Iwasaki et al. 2018 [46]	1
Yoshida et al. 2010 [47]	1
Yeo et al. 2013 [48]	1
Xiao et al. 2013 [49]	6
Wang et al. 2019 [50]	-

* Seven case reports were reviewed and analyzed by the authors; however, not referred in this review. ** The number of patients with V180I gCJD included in the article.

## Data Availability

Not applicable.
